# Corporate climate risk and public health disclosure: does digital transformation matter?

**DOI:** 10.3389/fpubh.2026.1954722

**Published:** 2026-09-23

**Authors:** Jiana Liu, Zhifeng Jie, Yan Wan

**Affiliations:** 1 Business School, Jiangxi Institute of Fashion Technology, Nanchang, China; 2 School of Tourism and Economic Management, Nanchang Normal University, Nanchang, China

**Keywords:** Chinese listed companies, corporate climate risk, digital transformation, public health disclosure, public health governance

## Abstract

**Introduction:**

Climate risk is playing an increasingly important role in shaping corporate behavior and non-financial disclosure. This study examines the nonlinear relationship between corporate climate risk (CR) and public health disclosure (PHD) using panel data from Chinese listed companies over the period 2000-2024.

**Methods:**

Empirically, this study employs a two-way fixed effects model, a moderating effect model, and a quantile regression model, accompanied by formal cross-quantile tests to evaluate the non-linear dynamics, moderating channels, and heterogeneity across conditional PHD distributions.

**Results:**

Four key findings emerge. First, CR exhibits a U-shaped relationship with PHD, and this relationship remains robust after a series of robustness and endogeneity checks. Second, conditional marginal effects and digital transformation (DT) specific turning points show that DT changes both the level and shape of the CR-PHD relationship, shifting the turning point to the right and attenuating the recovery of PHD at higher CR levels. Third, formal cross-quantile tests confirm that the CR and its quadratic term coefficients differ across the conditional PHD distribution. Fourth, the same tests identify significant differences across marketization and return on assets groups, whereas the differences across marginal profit groups are not statistically supported.

**Discussion:**

These findings highlight the role of corporate CR and technological capabilities in shaping PHD. The U-shaped dynamic indicates that moderate CR initially depresses PHD, whereas severe risk forces strategic transparency to restore stakeholder trust and corporate reputation. Furthermore, the moderating effect of DT underscores that digital capabilities buffer firms against immediate risk pressures, altering their disclosure thresholds. The observed heterogeneity across marketization levels and financial performance emphasizes the need for differentiated regulatory oversight that accounts for regional institutional environments and corporate resource slack in driving sustainable corporate transparency.

## 1 Introduction

Over the past several decades, China’s sustained economic expansion has substantially enhanced living standards, strengthened industrial capacity, and advanced societal well–being (1–3). Along with this progress, public health has become an increasingly important dimension of sustainable development. Public health is not only shaped by medical resources and health services, but also by environmental quality, economic stability, corporate behavior, and social governance capacity (4). However, climate risk has introduced new uncertainty into this process. Extreme weather events, temperature fluctuations, floods, droughts, and other climate related shocks may disturb production activities, increase environmental pressure, and weaken the stability of local economic and social systems (5, 6). These changes may further affect public health through employment, pollution exposure, resource allocation, and the resilience of public service systems. Against this background, this study investigates how corporate climate risk shapes firms’ public health related disclosure, with particular attention to the moderating role of digital transformation. The study provides new evidence for understanding the relationship between climate risk, corporate behavior, and health–related corporate disclosure.

As illustrated in Figure 1, the annual mean of corporate climate risk (CR) increased markedly between 2000 and 2024, with a particularly pronounced upward trend in recent years. This persistent increase suggests that climate related exposure has become an increasingly important concern for firms, thereby underscoring the need to examine its broader implications for the public health disclosure (PHD). The influence of CR on PHD can be understood through the close interdependence among firms, the environment, and society. Listed companies play important roles in economic production, resource utilization, technological innovation, and environmental governance (7, 8). Greater exposure to CR may alter firms’ production arrangements, operating costs, investment decisions, and risk-management strategies. In the short term, CR may generate adverse shocks by increasing production uncertainty, reducing operational efficiency, and intensifying environmental pressures, thereby weakening the socioeconomic foundations of public health (9). However, rising CR may also induce adaptive responses from firms and local governance systems. As climate related pressures intensify, firms may place greater emphasis on environmental responsibility, risk prevention, technological adoption, and resource use efficiency, while local governments and social organizations may strengthen climate adaptation and public health protection.

**Figure 1:**
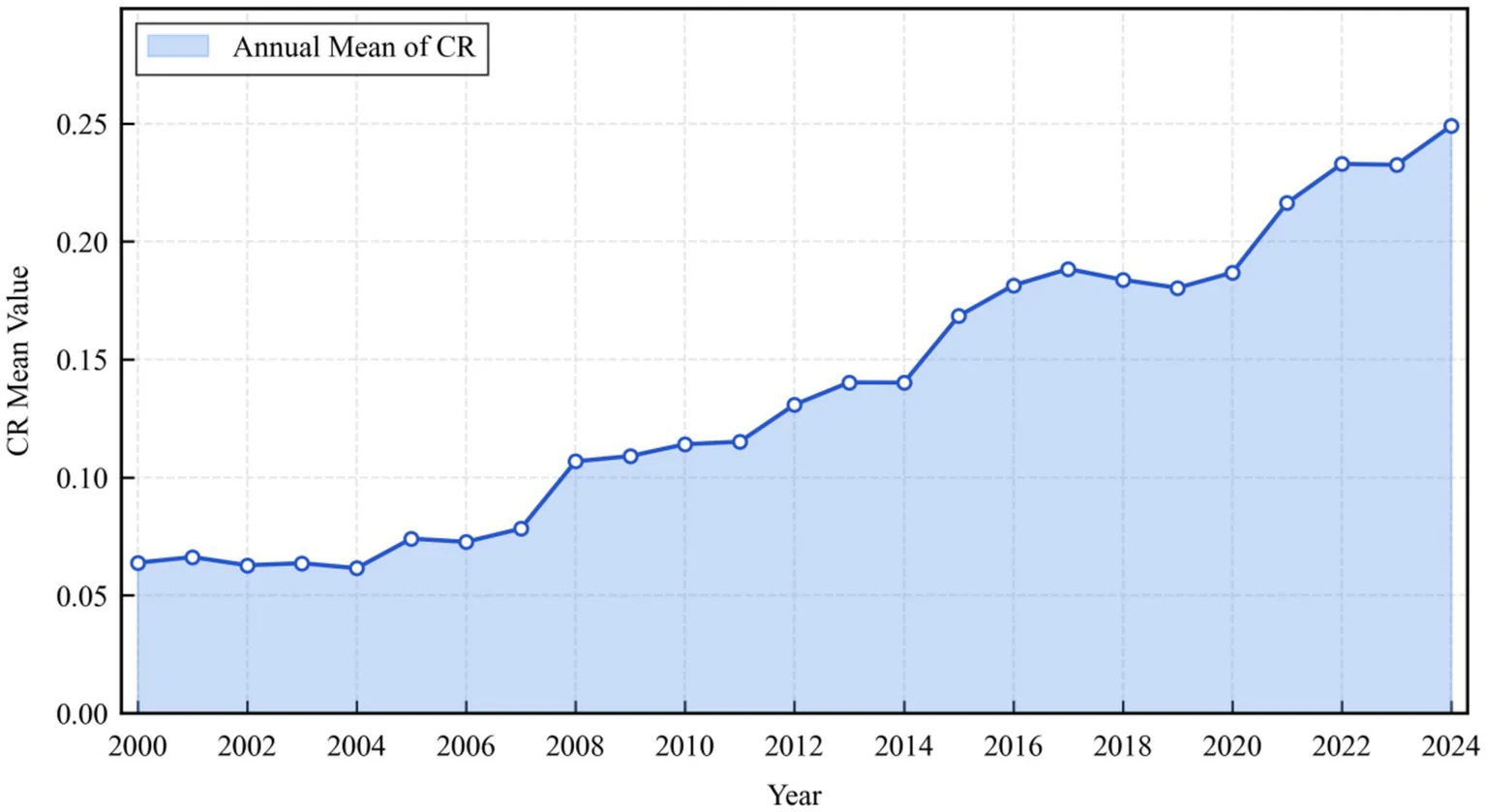
Annual average trend of CR index. This figure presents CR on its original scale before mean-centering.

PHD refers to the extent to which firms discuss health-related risks, responsibilities, and actions in their annual reports. It reflects the disclosed salience of these issues rather than objective population health outcomes (10–12). CR challenges these foundations. It raises important questions: Does CR simply damage PHD, or does its effect change across different levels of risk? Which factors moderate the relationship between CR and PHD? Under what conditions is the relationship between CR and PHD more significant? These questions are particularly important for China, where listed companies play a major role in economic development and where corporate health governance is closely connected with green transformation and corporate governance.

Existing research has provided useful insights into CR, corporate behavior, and PHD, but several limitations remain. First, many studies focus on the direct effects of climate change on health (13–15), while comparatively limited scholarly attention has been devoted to how firm–level climate risk affects corporate health related disclosure through corporate activities. Second, most studies assume a linear relationship between CR and social outcomes (16, 17). This may overlook the possibility that CR has different effects at different stages. In reality, moderate and severe CR may generate different responses from firms and local systems. Third, the role of corporate digital transformation has not been fully examined in the CR–PHD relationship. Digital transformation can improve information processing, monitoring capacity, production efficiency, and risk response (18, 19). However, whether it changes the nonlinear effect of CR on PHD still requires empirical testing. Finally, existing studies often ignore differences in PHD, market environments, and corporate profitability, which may lead to an incomplete understanding of the conditional effects of CR (20, 21).

This study seeks to bridge these research gaps by investigating the association between CR and PHD based on panel data from publicly listed Chinese firms, as illustrated in Figure 2. It develops a nonlinear analytical framework to investigate how the effect of CR on PHD. The framework considers that CR may initially adversely affect PHD by increasing operational uncertainty, environmental pressure, and resource constraints, while greater risk exposure may stimulate stronger adaptation, risk prevention, and governance responses. DT is further introduced as a moderating factor because digital technologies may enhance firms’ capacity to identify, monitor, and respond to climate-related risks. The empirical analysis examines whether DT changes the shape and intensity of the nonlinear relationship between CR and PHD. In addition, quantile regression model is further applied to determine whether the effect of CR differs across quantiles of the conditional PHD distribution. Sub–sample analyses are also conducted according to marketization level and firm financial characteristics, including return on assets and marginal profit rate, to examine how the institutional environment and corporate operating capacity shape the CR–PHD relationship.

**Figure 2:**
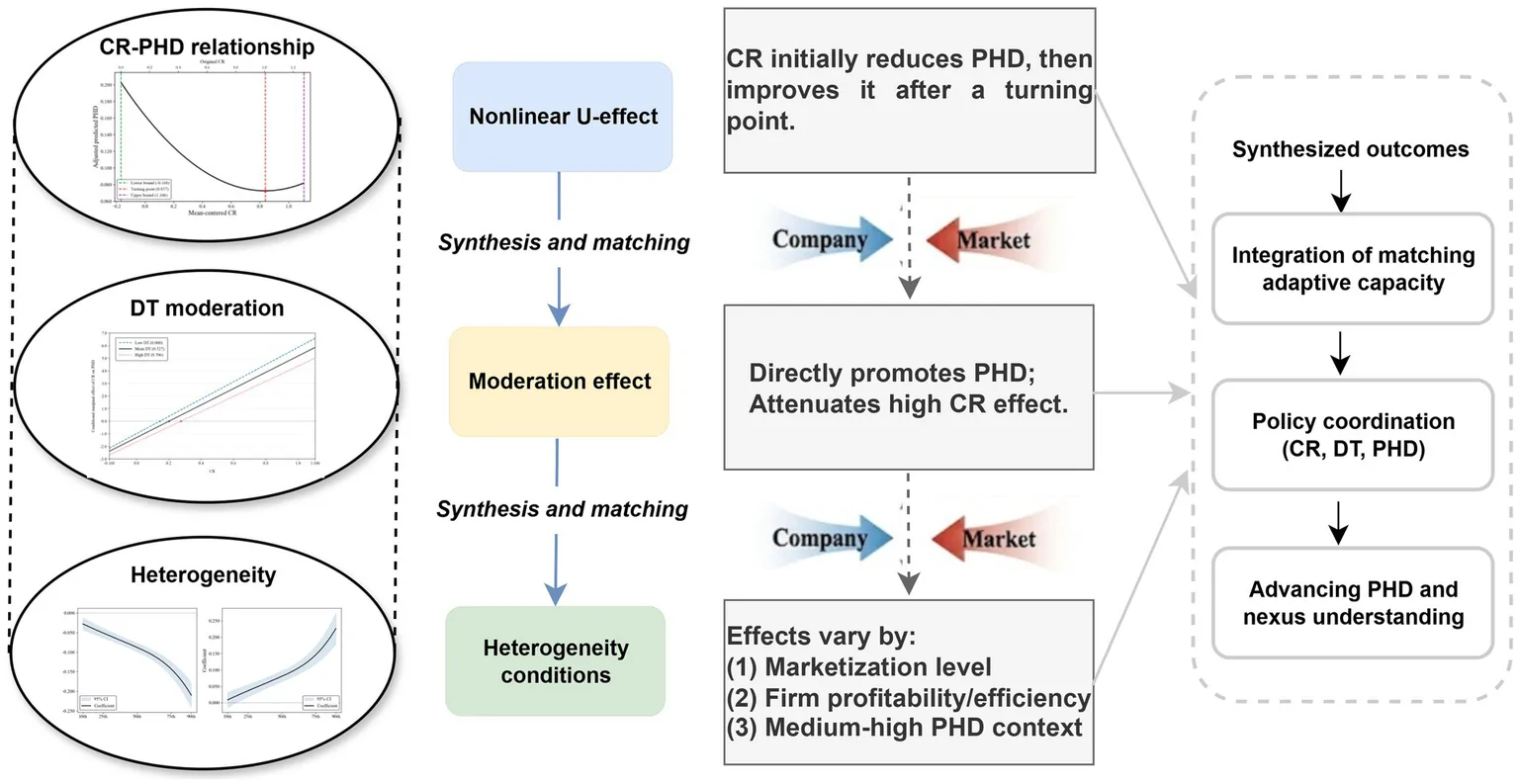
Research architecture diagram.

Ultimately, this research contributes to the literature in three ways. First, it extends CR research by linking firm–level CR with health-related corporate disclosure. This provides a new perspective for understanding the social consequences of CR. Second, it identifies a U–shaped relationship between CR and PHD, challenging the traditional linear view of CR effects. Third, it introduces corporate digital transformation as a moderating factor and shows that digital transformation negatively moderates the U–shaped relationship. These findings have important policy implications. CR governance should not focus only on environmental protection or disaster response, but should also consider its impact on PHD through corporate behavior. At the same time, promoting corporate digital transformation can improve risk response capacity and reduce the adverse consequences of CR. By revealing how CR affects PHD and how digital transformation changes this effect, this study provides useful evidence for climate governance, corporate transformation, and corporate health governance.

## 2 Literature review

Research on climate risk (CR) has developed along two largely separate lines. One strand examines its environmental and economic consequences, including production disruptions, financial losses, energy consumption, and disaster exposure (22–25). Another investigates the effects of climate related shocks on population health through air pollution, heat stress, infectious diseases, food security, and pressure on public services (26–28). Although these studies establish the importance of CR, they provide limited evidence on how firm level climate exposure shapes PHD. This distinction is important because PHD reflects the salience of health–related information in annual reports rather than objective population or regional health outcomes.

The literature also offers competing predictions regarding corporate disclosure responses to CR. From a resource constraint perspective, climate shocks increase operating uncertainty, production costs, and financial pressure, potentially crowding out managerial attention and expenditure devoted to health–related responsibilities (29). This view predicts a negative association between CR and PHD. By contrast, legitimacy and stakeholder perspectives suggest that increasingly material climate exposure attracts regulatory, investor, employee, and community scrutiny, strengthening firms’ incentives to disclose relevant risks and responses (30, 31). Research on climate adaptation similarly indicates that severe climate exposure prompt stronger risk management and organizational responses (32, 33). However, this literature does not establish whether these competing forces generate a constant disclosure response or whether their relative importance changes with the intensity of CR.

This unresolved debate raises the possibility of a nonlinear relationship. Most existing studies estimate an average linear effect and implicitly assume that the marginal influence of CR remains constant (34, 35). Such an assumption may conceal a transition between competing mechanisms. At relatively low CR levels, operational pressure and competing reporting priorities may reduce PHD, whereas sufficiently high CR may increase risk salience and external disclosure pressure. Theoretical arguments therefore permit a U–shaped association, but limited evidence has examined whether such a turning point exists in firm level public health related disclosure. Moreover, an increase in PHD beyond the turning point should be interpreted as a stronger disclosure response, not as direct evidence of successful adaptation or improved public health outcomes.

The role of digital transformation is similarly unresolved. Existing studies generally emphasize its capacity to improve information collection, risk identification, organizational coordination, and environmental monitoring (36–38). These capabilities may increase firms’ routine capacity to produce PHD. However, they may also allow firms to identify and communicate climate related risks earlier, reducing their reliance on sharp, reactive disclosure adjustments when CR becomes severe (39, 40). DT may therefore increase PHD at some CR levels while simultaneously weakening its marginal sensitivity to CR. Existing research has rarely distinguished this conditional level effect from DT’s moderating influence on the shape and turning point of the nonlinear CR–PHD relationship.

A further unresolved issue concerns heterogeneity. Institutional conditions, profitability, and information-processing capacity may influence firms’ ability and incentives to respond to climate risk (41, 42). Nevertheless, differences in coefficient significance across quantiles or separately estimated subgroups do not necessarily demonstrate statistically significant differences between them. Existing research provides limited formal evidence on whether the CR and CR^2^ coefficients differ across the conditional PHD distribution, marketization levels, or firms with different financial capacities.

Accordingly, this study addresses three related gaps. First, it examines the competing resource constraint and disclosure pressure explanations within a nonlinear CR–PHD framework. Second, it evaluates the role of DT through conditional marginal effects and DT–specific turning points rather than interpreting individual interaction coefficients in isolation. Third, it employs formal cross–quantile and cross–group tests to determine whether the nonlinear coefficients differ statistically across distributional, institutional, and firm level conditions.

## 3 Theoretical analysis and research hypothesis

### 3.1 The nonlinear impact of CR on PHD

Climate risk (CR) influences how firms allocate managerial resources and reporting attention to public health related issues. At relatively low levels of CR, increasing operational uncertainty, production costs, and supply chain disruptions may encourage firms to prioritize short–term operational stability and postpone environmental or health related expenditure (29, 43, 44). Because PHD measures the salience of health–related information in corporate annual reports, these competing pressures may crowd out managerial attention to such disclosure. Consequently, PHD may initially decline as CR increases. This decline represents a reduction in disclosure intensity rather than a deterioration in objective public health outcomes.

As CR becomes more severe, however, climate–related exposure is more likely to be regarded as a material corporate risk and attract greater attention from regulators, investors, employees, and local communities. Legitimacy theory suggests that firms facing heightened social and environmental scrutiny have stronger incentives to communicate their risks, responsibilities, and responses through corporate disclosure (30, 31). Previous research also indicates that severe climate exposure may prompt firms to strengthen risk management and consider adaptation responses (40, 45). In the present context, these pressures are expected to increase firms’ disclosure of occupational health, workplace safety, environmental health, emergency management, and protective activities. Therefore, PHD may rise after CR exceeds a certain threshold. This explanation concerns firms’ disclosure incentives and is consistent with, but does not directly demonstrate, actual adaptation investment, environmental improvement, or governance enhancement. Thus, we offer the following hypothesis.

*H1*: Corporate climate risk has a U–shaped association with corporate public health disclosure: PHD initially decreases as CR increases but rises after CR exceeds a certain threshold.

### 3.2 DT and the CR–PHL relationship

Digital transformation (DT) may affect PHD by improving firms’ capacity to collect, process, integrate, and communicate health and climate related information. Digital systems can reduce information–processing costs, strengthen internal coordination, and enable firms to identify relevant risks and disclosure requirements more efficiently (46, 47). At a given level of CR, firms with stronger digital capabilities may therefore disclose more information concerning occupational health, environmental health, workplace safety, and risk management.

The positive level effect of DT does not necessarily imply that DT strengthens the marginal disclosure response to every additional increase in CR. When climate risk becomes highly salient, stakeholder scrutiny and reporting pressure may already provide strong incentives for firms to increase PHD. Digitally transformed firms may also identify climate related risks earlier and incorporate related disclosure into routine information systems rather than responding through a sharp ex–post increase in disclosure. DT and climate risk pressure may therefore operate as partially substitutable drivers of PHD. Under this interpretation, DT can be positively associated with PHD at relatively low CR levels while reducing the additional increase in PHD induced by high CR (48). This attenuation concerns the sensitivity of corporate disclosure to CR and does not imply that DT weakens firms’ actual climate adaptation capacity. Thus, we offer the following hypothesis.

*H2*: DT conditionally moderates the U–shaped association between CR and PHD. Higher DT shifts the turning point and weakens the positive marginal effect of CR on PHD at relatively high CR levels.

### 3.3 Heterogeneous effects across different levels of PHD

The impact of CR on PHD may vary across different levels of public health conditions. PHD reflects the extent to which firms disclose health–related risks, responsibilities, and protective actions in their annual reports, rather than actual health outcomes or regional public–health capacity (49, 50). When PHD is low, local systems may face constraints in reporting systems, stakeholder scrutiny, ESG governance capacity and information-processing capability. Firms and governments may have limited ability to convert climate pressure into effective adaptation. Under such conditions, the nonlinear relationship between CR and PHD may be weak or difficult to observe.

In contrast, when PHD is at a medium or high level, the response mechanism may be more complete. Regions or contexts with better public health foundations may have stronger monitoring capacity, better public services, and more effective governance systems (51). Firms operating in these environments may also face stronger social expectations and regulatory pressure. As a result, the negative effect of lower CR and the adaptive effect of higher CR may both be more visible. This may make the U–shaped relationship between CR and PHD more significant at medium and high levels of PHD.

This difference suggests that the CR–PHD relationship should not be understood only from the average effect. The same level of CR may generate different outcomes under different public health conditions. Panel quantile analysis can therefore help identify whether the nonlinear effect changes across the PHD distribution. In this regard, we propose the following hypothesis.

*H3*: The U–shaped relationship between CR and PHD is more pronounced at the middle and upper quantiles of the PHD distribution.

### 3.4 Heterogeneity effects of marketization level, return on assets, and marginal profit rate

Marketization level, return on assets, and marginal profit rate create different external and internal conditions for firms to respond to CR. The market environment affects resource allocation, competition pressure, information transparency, and the efficiency of institutional constraints (52, 53). In regions with higher marketization levels, firms may face stronger market discipline and clearer incentives for risk management. They may also have better access to capital, technology, and professional services. These conditions can improve firms’ ability to respond to CR and make the nonlinear relationship between CR and PHD more evident.

Corporate profitability further shapes this process. Return on assets serves as an indicator of firms’ asset-utilization efficiency and overall operational performance (54, 55). Firms with higher return on assets may have stronger capacity to absorb climate shocks, maintain production stability, and invest in environmental or digital management. Similarly, marginal profit rate reflects firms’ profit space and cost-bearing capacity (56). Firms with higher marginal profit rates may have more room to adjust production, improve risk management, and support long term adaptation. These advantages may strengthen the transmission from CR to adaptive behavior and make the U–shaped relationship more significant. In contrast, firms facing lower marketization conditions or weaker profitability may have limited adjustment capacity. They may respond to CR mainly through short-term cost control rather than long-term adaptation. This may weaken the nonlinear effect of CR on PHD. Therefore, the CR–PHD relationship depends not only on climate pressure itself, but also on the institutional and financial conditions under which firms operate. In this regard, we propose the following hypotheses.

*H4*: The U–shaped relationship between CR and PHD exhibits heterogeneity across different levels of marketization.

*H5*: The U–shaped relationship between CR and PHD exhibits heterogeneity across firms with different levels of return on assets.

*H6*: The U–shaped relationship between CR and PHD exhibits heterogeneity across firms with different marginal profit rates.

## 4 Methodology, variable, and data

### 4.1 Methodology

This study adopts an integrated empirical strategy to examine the nonlinear effect of CR on PHD. Fixed effects panel regressions are used to identify the baseline relationship and control to account for unobservable firm specific and time varying effects. The moderating role of DT is then examined by incorporating nonlinear interaction structures into the model. In addition, panel quantile regressions are employed to assess whether the CR–PHD relationship varies across different levels of corporate public health disclosure.

#### 4.1.1 Baseline model specification

The baseline specification is estimated using a fixed effects panel model, which helps account for unobservable firm level characteristics and common time shocks (57, 58). Drawing on the theoretical link between climate pressure, corporate behavior, and health–related corporate disclosure, the model is constructed to examine whether the effect of CR on PHD follows a U–shaped pattern. The basic regression equation is specified as the Equation 1:

$$\mathrm{PH}D_{\mathrm{it}}=\alpha +\beta_{1}CR_{\mathrm{it}}+\beta_{2}CR_{\mathrm{it}}^{2}+\text{θContro}l_{\mathrm{it}}+\mu_{i}+\mu_{t}+\varepsilon_{\mathrm{it}}$$ (1)

Where $\mathrm{PH}D_{\mathrm{it}}$ is the public health disclosure in year t for firm i . $CR_{\mathrm{it}}$ expresses the corporate climate risk. $\text{Contro}l_{\mathrm{it}}$ is the control variable vector. $\mu_{i}$ and $\mu_{t}$ represent firm and year fixed effects, respectively, to account for time–invariant firm characteristics.

#### 4.1.2 Moderator model specification

To test whether DT moderates the CR–PHD relationship, the model introduces interaction terms between DT variables and CR as the Equation 2.

$$\begin{array}{l} \mathrm{PH}D_{\mathrm{it}}=\alpha +\beta_{1}CR_{\mathrm{it}}+\beta_{2}CR_{\mathrm{it}}^{2}+\beta_{3}(CR_{\mathrm{it}}\times DT_{\mathrm{it}})+\\ \beta_{4}(CR_{\mathrm{it}}^{2}\times DT_{\mathrm{it}})+\beta_{5}DT_{\mathrm{it}}+\\ \text{θContro}l_{\mathrm{it}}+\mu_{i}+\mu_{t}+\varepsilon_{\mathrm{it}} \end{array}$$ (2)

Where $DT_{\mathrm{it}}$ represents digital transformation. The coefficients $\beta_{3}$ and $\beta_{4}$ capture the extent to which DT modifies the gradient and curvature of the U–shaped relationship.

#### 4.1.3 Quantile regression model specification

Further, the theoretical analysis and research hypotheses suggest that the relationship between CR and PHD is not only nonlinear, but may also vary across different levels of PHD. The fixed–effects model discussed above estimates the average effect of the variables within a conditional mean framework, and therefore cannot fully capture the distributional differences and heterogeneous effects under different PHD conditions. The quantile regression approach proposed by Koenker and Hallock (59) provides a useful tool for identifying potential nonlinear and asymmetric relationships among variables. Accordingly, this study extends the analysis to an asymmetric framework and employs a panel quantile regression model to further examine how the effect of CR on PHD differs across the distribution of PHD. The conditional quantile of the random variable is specified as the Equation 3:

$$\mathrm{PH}D_{\mathrm{it}}=\sigma_{i}+CR_{\mathrm{it}}^{`}\tau +(\lambda_{i}+Q_{\mathrm{it}}^{`}\delta )R_{\mathrm{it}}$$ (3)

In the above equation, σ , τ , λ , and δ are estimated parameters satisfying $P(\lambda_{i}+Q_{\mathrm{it}}^{`}>0)$ =1. $R_{\mathrm{it}}$ denotes an unobserved random vector that is independently and identically distributed across individuals and is orthogonal to $CR_{\mathrm{it}}$ . It also satisfies E(∣R∣) = 1 and E(R) = 0. Further expansion gives in the Equation 4:

$$\mathrm{PH}D_{y}(\partial |CR_{\mathrm{it}})=[\sigma_{i}+\lambda_{i}m(\partial )]+CR_{\mathrm{it}}^{`}\tau +Q_{\mathrm{it}}^{`}\mathrm{\delta m}(\partial )$$ (4)

Where $\mathrm{PH}D_{y}(\partial |CR_{\mathrm{it}})$ represents the quantile distribution of the dependent variable, $y_{\mathrm{it}}\sigma_{i}(\partial )\equiv \sigma_{i}+\varepsilon_{i}m(\partial )$ denotes a scalar coefficient, and the remaining variables are defined as above.

### 4.2 Variable description

#### 4.2.1 Data source

The empirical analysis draws on a sample of Chinese publicly listed companies covering the period from 2000 to 2024. Firm level data are obtained from the China Stock Market and Accounting Research (CSMAR) database and the Wind database. Corporate PHD and CR are measured using data collected through web scraping. To ensure data quality, the sample excludes ST, ^*^ST, and PT firms, suspended firms, and observations with substantial missing values with the core variables. Except for ratio variables, all variables are logarithmically transformed. A small number of missing observations are filled using linear interpolation. To reduce the influence of outliers caused by financial anomalies, reporting errors, or exceptional events, all continuous variables are winsorized at the 1st and 99th percentiles.

#### 4.2.2 Dependent variable

The dependent variable is corporate public health disclosure (PHD). The textual corpus consists of the annual reports of Chinese listed firms, primarily obtained from CNINFO and the official websites of the Shanghai, Shenzhen, and Beijing Stock Exchanges. Missing reports are supplemented using documents published on the official websites of the corresponding firms. Reports that are incomplete, illegible, or cannot be reliably converted into machine–readable text are excluded. Following He et al. (60) and Liu et al. (61), we construct PHD through textual analysis of the collected annual reports. The dictionary was developed by reviewing terminology used in annual reports, corporate social responsibility reports, ESG reports, and relevant studies. Supplementary Table S1 reports the complete keyword list and contextual classification rules.

PHD is measured as the number of validated public health related expressions per 10,000 words in each annual report. It captures the intensity and salience of corporate disclosure concerning health–related risks, responsibilities, and actions rather than objective population or regional public health outcomes. To clarify the directional meaning of the construct, we further distinguish adverse health–risk disclosure (AHRD) from protective health–action disclosure (PHAD). Directionally ambiguous expressions are retained in aggregate PHD but excluded from AHRD and PHAD. A higher PHD value therefore indicates more intensive public health related disclosure, whereas higher AHRD and PHAD, respectively, indicate greater disclosure of adverse health risks and protective health actions.

#### 4.2.3 Independent variable

The independent variable is corporate climate risk (CR). Following Li et al. (62) and Li and Yao (63), we construct the CR measure using textual analysis of the annual reports of Chinese A–share listed firms obtained from the CNINFO database. Scanned reports and reports containing incomplete information are excluded. A CR dictionary adapted to the Chinese institutional and corporate reporting context is then developed, and the initial CR index is calculated as the frequency of CR related terms relative to the total number of words in each annual report. Physical-risk terms cover acute extreme-weather events and chronic climatic changes, whereas transition-risk terms capture climate-policy, regulatory, market, technological, and reputational pressures. Supplementary Table S2 reports the complete set of English equivalents of the proposed search terms. To reduce false-positive matches, general transition-related expressions are counted only when they occur in a sentence containing a risk-context cue, such as risk, pressure, uncertainty, exposure, loss, cost, constraint, compliance, penalty, impact, threat, or vulnerability. CR is measured as the number of qualified climate-risk expressions per 10,000 words. A higher value indicates greater disclosed exposure to and salience of corporate CR. The initial CR index is mean-centered before its quadratic term is constructed for the empirical analysis.

A higher value indicates greater disclosed exposure and attention to climate related risks. For the empirical analysis, the initial CR index is mean–centered before constructing its quadratic term. Accordingly, negative values of the centered CR variable indicate below average climate risk exposure rather than negative climate risk. This transformation ensures that the descriptive statistics, regression estimates, U–test, and nonlinear graphical analysis are presented on a consistent scale. Figure 3 illustrates the annual distribution and temporal evolution of the CR index before centering. As mean–centering changes only the location of the distribution without altering its dispersion, relative ordering, or temporal pattern, the figure remains directly informative about the evolution of corporate climate risk exposure. The results show that CR generally increased between 2000 and 2024, with more pronounced growth after 2010 and increasing dispersion across firms.

**Figure 3:**
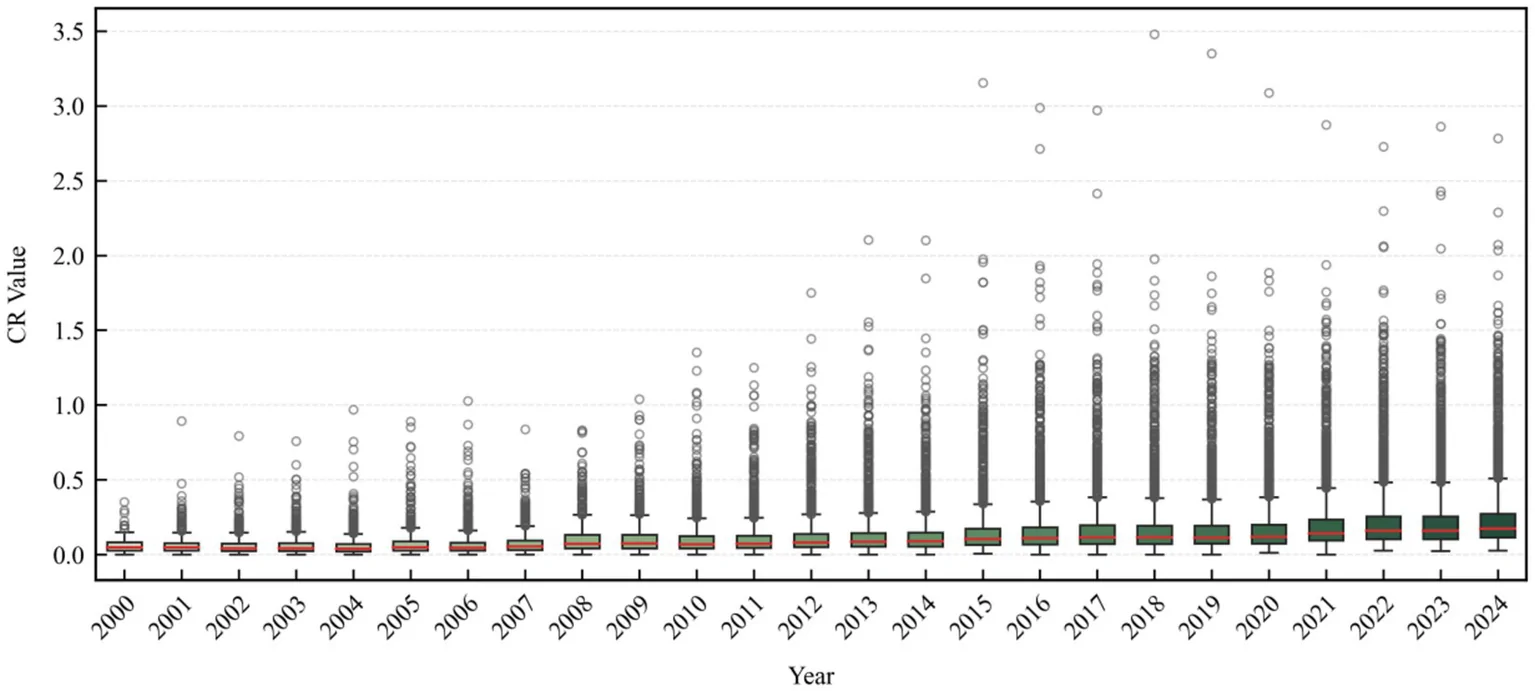
Annual distribution and evolution of CR index. This figure presents CR on its original scale before mean-centering.

#### 4.2.4 Moderating variable

Corporate digital transformation (DT) is the moderating variable. Following Chen et al. (64), Sui et al. (65), and Zhang and Jie (66), the proposed DT dictionary covers three dimensions: digital technologies, digital organizational empowerment, and digital business applications. Supplementary Table S3 reports all 139 English equivalents of the proposed search terms. The matching procedure applies a longest–match rule so that nested expressions are not counted twice. For example, an occurrence of digital transformation is not counted again as a separate occurrence of digitalization. DT is measured as the number of validated digital transformation expressions per 10,000 words in each annual report. A higher value indicates greater disclosed intensity and strategic emphasis on corporate DT.

#### 4.2.5 Control variables

Following prior research by Deng and Jie (67), this study controls for other firm characteristics, including firm growth (FG), executive incentives (EI), cash flow status (CFS), firm size (FS), debt ratio (DR), return on assets (ROA), net assets per share (NAP), and fixed asset ratio (FAR). Detailed variable definitions are reported in Table 1, and the descriptive statistics are presented in Table 2.

**Table 1:** Variable definitions.

Variable type	Definition	Measurement	Symbol
Dependent variable	Public health disclose	Frequency of public health-related terms	PHD
Independent variable	Climate risk	Frequency of climate risk-related terms divided by the total word count	CR
Moderating variable	Digital transformation	Frequency of digital transformation-related keywords	DT
Control variable	Firm growth	Growth rate of operating revenue	FG
	Executive incentives	Executive shareholdings divided by the total number of shares outstanding	EI
	Cash flow status	The ratio of net cash flows generated from operating activities to total assets	CFS
	Firm size	The year-end value of total assets expressed in natural logarithmic form	FS
	Debt ratio	Total liabilities divided by total assets at year-end	DR
	Return on assets	The ratio of net income to total assets measured at the end of the fiscal year	ROA
	Net assets per share	The ratio of shareholders’ equity to the total number of outstanding shares	NAP
	Fixed asset ratio	Net fixed assets divided by total assets	FAR

**Table 2:** Descriptive statistics.

Variable	Mean	P50	Min	Max	Sd	N
PHD	0.163	0.136	0.019	0.704	0.108	28,263
CR	0.000	−0.060	−0.168	1.106	0.190	37,590
DT	0.327	0.208	0.000	2.541	0.379	37,590
FG	0.001	0.001	−0.309	0.444	0.075	37,590
EI	0.055	0.039	0.001	0.458	0.053	37,590
CFS	0.249	0.238	−2.197	1.605	0.418	36,044
FS	21.859	21.682	4.605	30.031	1.402	37,590
DR	0.545	0.545	0.544	0.725	0.002	37,585
ROA	0.585	0.596	−0.397	0.808	0.075	28,876
NAP	4.654	4.645	4.303	5.295	0.040	37,586
FAR	0.065	0.066	0.034	0.167	0.001	37,586

## 5 Empirical section

### 5.1 Benchmark regression

This study employs panel regression models with firm and year fixed effects to examine the nonlinear impact of corporate climate risk (CR) on public health level (PHD). The regression results are reported in Table 3. Model specifications progressively expand: Model (1) estimates the baseline linear effect of CR without additional covariates, Models (2)–(5) progressively introduce firm-level covariates to improve the reliability of the estimated results, and Model (6) further introduces a quadratic term to capture potential nonlinearity. Across Models (1)–(5), the coefficient of CR remains negative and statistically significant, indicating that increasing CR is initially associated with lower public health disclosure intensity. This result should not be interpreted as evidence that CR directly worsens regional or population health. Model (6) further reveals a U–shaped relationship between corporate CR and PHD. The linear coefficient of CR turns to −0.216 and remains significantly negative, while the quadratic coefficient of CR2 is significantly positive at 0.129, jointly identifying a structural inflection point in the relationship. Economically, moderate levels of CR initially depress PHD through direct harm mechanisms, whereas at sufficiently elevated CR levels, compensatory responses such as strengthened regulatory scrutiny, accelerated green transition, and expanded adaptive investment gradually attenuate the adverse effects (40, 44, 45), thereby generating the upward–turning segment of the curve. The statistical significance of the quadratic term formally rejects the linear specification in favor of a nonlinear functional form, providing empirical support for the existence of a threshold beyond which the marginal effect of CR on public health reverses in direction.

**Table 3:** Benchmark regression results.

	(1)	(2)	(3)	(4)	(5)	(6)
CR	−0.055^***^ (0.005)	−0.021^***^ (0.004)	−0.024^***^ (0.004)	−0.024^***^ (0.004)	−0.028^***^ (0.004)	−0.216^***^ (0.007)
FG		0.067^***^ (0.011)	0.062^***^ (0.011)	0.058^***^ (0.011)	0.061^***^ (0.010)	0.058^***^ (0.010)
EI		1.520^***^ (0.012)	1.447^***^ (0.012)	1.423^***^ (0.012)	1.242^***^ (0.012)	1.161^***^ (0.012)
CFS			0.053^***^ (0.002)	0.053^***^ (0.002)	−0.004^*^ (0.002)	−0.003 (0.002)
FS			0.000 (0.000)	0.000 (0.000)	−0.000^***^ (0.000)	−0.000^***^ (0.000)
DR				0.005^***^ (0.000)	0.005^***^ (0.000)	0.005^***^ (0.000)
ROA				−0.024^***^ (0.004)	0.008^***^ (0.000)	0.008^***^ (0.000)
NAP					0.001^***^ (0.000)	0.001^***^ (0.000)
FAR					−0.028^***^ (0.004)	0.265^***^ (0.010)
CR^2^						0.129^***^ (0.007)
$\mu_{i}$	Yes	Yes	Yes	Yes	Yes	Yes
$\mu_{t}$	Yes	Yes	Yes	Yes	Yes	Yes
*Adj R^2^*	0.281	0.548	0.561	0.564	0.596	0.609
*N*	28,263	28,263	27,151	27,151	27,151	27,151

Standard errors are reported in parentheses. ^*^p < 0.1, ^**^p < 0.05, ^***^p < 0.01.

Following the U–test approach proposed by Lind and Mehlum (68), this study further examines the nonlinear relationship between corporate CR and PHL. The U–test results reported in Table 4 formally verify the U–shaped relationship by evaluating the marginal effects at the boundary values of the sample distribution. At the lower bound of the CR range (−0.168), the estimated slope is −0.259 with a t–statistic of −6.225, statistically significant at the 1% level, indicating that within the low–CR region, additional CR exposure produces a significantly negative marginal effect on PHL. At the upper bound of the CR range (1.106), the slope reverses to 0.069 with a t-statistic of 3.170, and remains statistically significant. This finding indicates that, under high CR conditions, compensatory mechanisms increasingly outweigh the direct adverse channels, resulting in a significantly positive marginal effect. The extreme point of the fitted curve is identified at CR = 0.837, precisely pinpointing the inflection value at which the marginal effect of corporate climate risk on public health transitions from negative to positive. The overall t–value of 4.114 with a corresponding *p*–value of 0.000 substantially exceeds conventional critical thresholds, decisively rejecting linear or inverted U–shaped alternatives in favor of the U–shaped functional form.

**Table 4:** U-test test of CR on PHL.

Type	Lower bound	Upper bound
Value range	−0.168	1.106
Slope	−0.259	0.069
T-value	−6.225	3.170
*P*-value	0.000	0.000
Extreme point	0.837	
Overall t-value	4.114	
Overall *p*-value	0.000	

Figure 4 depicts the nonlinear trajectory derived from Model (6), which visually illustrates that public health level declines steadily as CR increases from −0.168 to the inflection point of 0.837, and subsequently rises as CR continues to expand toward 1.106, thereby exhibiting a well–defined convex pattern centered on the identified turning point. Taken together, the U–test results empirically substantiate the proposed U–shaped nexus between corporate CR and PHL, thereby lending robust support to *Hypothesis 1*. Because PHD measures disclosure intensity, the rising portion of the curve should be interpreted as an increase in PHD rather than an improvement in population health or direct evidence of successful climate adaptation.

**Figure 4:**
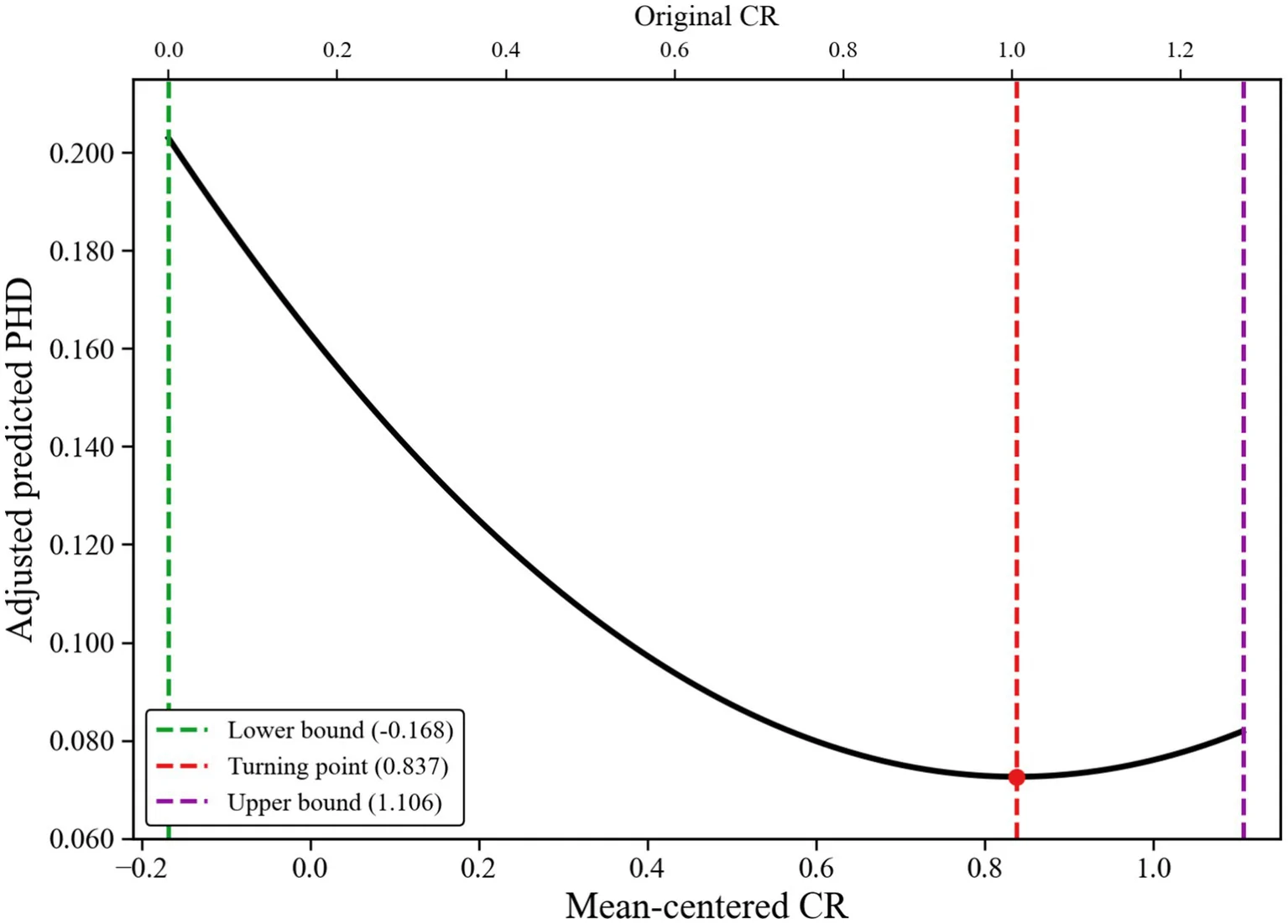
Adjusted nonlinear relationship between CR and PHD.

### 5.2 Robust tests

To assess the sensitivity of the baseline results to potential endogeneity arising from omitted variables and reverse causality, this study conducts a two–stage least squares (2SLS) analysis (69, 70). Specifically, the one–period lagged values of firm level CR and CR^2^ are used as instruments for their contemporaneous values. The relevance of these instruments is based on the persistence of firms’ climate risk exposure: past CR contains information that strongly predicts current CR, while contemporaneous PHD cannot affect previously realized climate risk exposure.

Nevertheless, instrument relevance does not by itself establish the exclusion restriction. Lagged CR may remain correlated with persistent unobserved firm characteristics or may affect current PHD through channels extending across reporting periods. Accordingly, the lagged variable strategy cannot completely rule out reverse causality or omitted variable bias. We therefore treat this analysis as a supplementary endogeneity check rather than as definitive causal identification.

The results are presented in Table 5. In the first stage, lagged CR is strongly associated with current CR, with a coefficient of 0.999 that is significant at the 1% level. Lagged CR^2^ is also strongly associated with current CR^2^, with a coefficient of 0.920 and significance at the 1% level. The Kleibergen–Paap LM statistic of 2100.04 rejects the null hypothesis of under–identification. Moreover, the Cragg–Donald Wald F statistic of 5100.27 substantially exceeds the relevant critical value of 7.03, indicating that weak instrument relevance is unlikely to be a material concern.

**Table 5:** Endogeneity check using CR lagged by one period as the instrumental variable.

2SLS estimation	First stage		Second stage
	CR	CR^2^	PHD
L. CR	0.999^***^ (0.003)	0.069^***^ (0.003)	
L. CR^2^	−0.016^***^ (0.005)	0.920^***^ (0.004)	
CR (IV)			−0.114^***^ (0.007)
CR^2^ (IV)			0.113^***^ (0.010)
Control variables	Yes	Yes	Yes
$\mu_{i}$	Yes	Yes	Yes
$\mu_{t}$	Yes	Yes	Yes
*N*	26,951	26,951	26,951
Adj. *R*^2^	0.914	0.893	0.084
Kleibergen-Paap LM statistic		2100.04^***^	
Cragg-Donald Wald F statistic		5100.27 > 7.03	

Standard errors are reported in parentheses. ^*^p < 0.1, ^**^p < 0.05, ^***^p < 0.01.

In the second stage, the coefficient of instrumented CR is significantly negative, whereas that of instrumented CR^2^ is significantly positive. Specifically, the coefficients of CR (IV) and CR^2^ (IV) are −0.114 and 0.113, respectively, and both are significant at the 1% level. These estimates are consistent with the U–shaped association identified in the baseline analysis: the intensity of public-health-related corporate disclosure initially decreases with CR but increases after CR exceeds a certain threshold. The lagged–IV estimates therefore provide supportive robustness evidence for the baseline nonlinear relationship. Given the limitations of the exclusion restriction, however, they should not be interpreted as independently eliminating all concerns regarding reverse causality and omitted variables. We consequently supplement this analysis with an external instrumental variable strategy based on data independent of corporate annual reports.

To address potential endogeneity arising from reverse causality, omitted variables, and the persistence of annual report based textual measures, we replace the lagged CR instruments with an external climate–salience (ECS) instrument. The exposure component is constructed using historical meteorological conditions in the firm’s registered city during the pre–sample period of 1981–1999. Firm locations are obtained from the CSMAR database and matched with daily observations from the China Meteorological Data Service Centre. For each city, we calculate the average annual frequencies of extreme temperature days, heavy precipitation days, and prolonged dry spells relative to the city specific historical distribution. These indicators are standardized and combined into a historical climate exposure index. Because this exposure measure is determined before the estimation period, it is not affected by firms’ subsequent climate risk or public health disclosure decisions.

The external shock component is based on the global climate policy uncertainty index provided by the ISETS Energy Finance Network. We calculate an annual leave-China-out index using the climate policy uncertainty indices of the other countries covered by the database and use its annual change to capture external climate-information shocks. The instrumental variable is constructed by interacting each firm’s predetermined historical climate exposure with the annual leave-China-out climate-policy uncertainty shock. This construction produces firm level differences in exposure to a common external shock while remaining independent of the textual content of Chinese corporate annual reports. The instrument and its squared term are used as excluded instruments for CR and CR^2^, respectively. All specifications include the baseline control variables and firm and year fixed effects.

Table 6 reports the relevant results. The first-stage results demonstrate that the external instruments are strongly related to the two endogenous CR terms. ECS is positively associated with CR, with a coefficient of 0.096, while ECS^2^ is strongly associated with CR^2^, with a coefficient of 0.074. The Kleibergen–Paap rk LM statistic rejects the null hypothesis of under–identification at the 1% level, and Cragg–Donald Wald F statistic is 41.63, which is far above the critical value of 7.03, providing no indication of a material weak-instrument problem. In the second stage, the instrumented coefficient of CR is negative and statistically significant, whereas the coefficient of CR^2^ is positive and statistically significant, supporting the presence of a U–shaped relationship.

**Table 6:** Endogeneity check using external climate-salience instrument as the instrumental variable.

2SLS estimation	First stage		Second stage
	CR	CR^2^	PHD
ECS	0.096^***^ (0.013)	0.014^**^ (0.007)	
ECS^2^	0.028^***^ (0.010)	0.074^***^ (0.011)	
CR (IV)			−0.247^***^ (0.041)
CR^2^ (IV)			0.151^***^ (0.028)
Control variables	Yes	Yes	Yes
$\mu_{i}$	Yes	Yes	Yes
$\mu_{t}$	Yes	Yes	Yes
*N*	27,151	27,151	27,151
Adj. *R*^2^	0.512	0.438	0.566
Kleibergen-Paap LM statistic		58.72^***^	
Cragg-Donald Wald F statistic		41.63 > 7.03	

Standard errors are reported in parentheses. ^*^p < 0.1, ^**^p < 0.05, ^***^p < 0.01.

### 5.3 Moderate role of digital transformation

Building on the nonlinear association between CR and PHD, this section examines whether DT changes the shape of this relationship. Because the model contains interactions between DT, CR, and CR^2^, neither the DT coefficient nor either interaction coefficient can be interpreted in isolation. We evaluate the moderation effect using a joint test of the interaction terms, the conditional marginal effects of DT and CR, and DT–specific turning points. The regression coefficients are reported in Table 7, while the corresponding conditional results are presented in Table 8.

**Table 7:** Regression outcomes of the moderating effect of DT.

	(1)	(2)	(3)
CR	−2.492^***^ (0.294)	−0.988^***^ (0.251)	−1.484^***^ (0.276)
CR^2^	9.750^***^ (1.269)	3.428^***^ (1.072)	5.344^***^ (1.166)
DT	0.907^***^ (0.149)	0.324^**^ (0.127)	0.569^***^ (0.139)
DT × CR	−3.327^***^ (0.639)	−0.954^**^ (0.474)	−1.897^***^ (0.586)
DT × CR^2^	−1.183^***^ (0.017)	−0.579^***^ (0.015)	−0.590^***^ (0.016)
Control variables	No	Yes	Yes
$\mu_{i}$	Yes	Yes	Yes
$\mu_{t}$	Yes	Yes	Yes
*Adj R^2^*	0.462	0.630	0.611
*N*	28,263	27,151	28,142

Standard errors are reported in parentheses. ^*^p < 0.1, ^**^p < 0.05, ^***^p < 0.01.

**Table 8:** Conditional marginal effects and DT-specific turning points.

Panel A. Conditional marginal effect of DT	Estimate	SE	95% CI	*p*-value
CR lower bound (−0.168)	0.468	0.110	[0.252, 0.684]	<0.001
CR median (−0.063)	0.382	0.095	[0.196, 0.568]	<0.001
Turning region for mean DT (0.201)	0.109	0.080	[−0.048, 0.266]	0.172
CR upper bound (1.106)	−1.439	0.250	[−1.929, −0.949]	<0.001

Panel B. DT-specific nonlinear profile	Curvature	Turning point [95% CI]	Lower-bound slope	Upper-bound slope	U-test *p*
Low DT (0.000)	3.428^***^	0.144 [0.097, 0.191]	−2.140^***^	6.594^***^	<0.001
Mean DT (0.327)	3.239^***^	0.201 [0.148, 0.253]	−2.388^***^	5.864^***^	<0.001
High DT (0.706)	3.019^***^	0.275 [0.209, 0.342]	−2.676^***^	5.017^***^	<0.001
Joint test	Wald chi-square	1494.21 *p* < 0.001			

^*^p < 0.1, ^**^p < 0.05, ^***^p < 0.01.

The coefficients of CR are significantly negative and those of CR^2^ are significantly positive across the specifications. In Column (2), the coefficient of DT is 0.324 and statistically significant at the 5% level. However, this coefficient represents the association between DT and PHD only when CR equals zero. It should not be interpreted as evidence that DT unconditionally improves PHD across the entire CR distribution. The substantive effect of DT depends jointly on CR, CR^2^, and their interaction terms. The coefficients of DT × CR and DT × CR^2^ are negative in the preferred specification. This finding provides support for *Hypothesis 2*. These estimates suggest that higher DT lowers the conditional marginal effect of CR and reduces the curvature of the CR–PHD relationship. Nevertheless, the direction and significance of the moderation effect cannot be determined from these coefficients separately. We therefore rely on the conditional effects and DT–specific nonlinear profiles reported in Table 8 to interpret the results.

Column (3) of Table 7 further replaces the measurement of moderator variable, DT, to examine the moderating effect robustness. For the measurement of firms’ DT, most studies adopt text analysis methods. Following prior studies (71), this study uses the number of patent applications at the company level to measure the degree of digital transformation. This alternative measure of DT is subsequently used to replace the original explanatory variable for robustness verification. The results remain consistent. CR remains significantly negative, CR^2^ remains significantly positive, DT is significantly positive, and both interaction terms retain negative and statistically significant coefficients. This confirms that the moderating effect of DT is not dependent on a particular measurement of PHD.

Panel A of Table 8 reports the conditional marginal effect of DT calculated from the actual interaction coefficients. The point estimate is positive at the lower bound of CR (0.468) and at the median (0.382), becomes smaller around the turning region for mean DT (0.109), and is negative at the upper bound (−1.439). Thus, the association between DT and PHD varies across the CR distribution: DT is associated with greater PHD at relatively low CR levels but attenuates the disclosure response when CR is high.

Panel B of Table 8 evaluates the nonlinear profile at low DT (the observed lower support of zero), mean DT (0.327), and high DT (one standard deviation above the mean, 0.706). Based on the actual coefficients in Column (2), the conditional curvature declines from 3.428 to 3.239 and 3.019 as DT increases. The corresponding turning point shifts from 0.144 to 0.201 and 0.275 on CR scale. The lower–bound slope becomes more negative, whereas the positive upper–bound slope becomes smaller. This conditional pattern indicates that DT shifts the turning point to the right and attenuates the upward branch of the U–shaped relationship.

The conditional results help explain the apparently counter–intuitive findings. DT is positively associated with PHD at relatively low CR levels, which is consistent with its information–processing and disclosure capacity advantages. As CR increases, however, the marginal effect of DT becomes smaller, while higher DT shifts the U–shaped turning point to the right and reduces the positive upper branch slope. This pattern suggests that digitally transformed firms rely less on sharp, risk-induced increases in disclosure because climate- and health-related information may already be incorporated into their routine reporting systems. Thus, DT raises disclosure capacity under relatively low CR but attenuates the incremental disclosure response to high CR. This is a stabilization or substitution effect in corporate reporting rather than evidence that DT weakens substantive climate adaptation.

Figure 5 plots the conditional marginal effect of CR on PHD at the same three DT levels using the actual coefficients from Column (2) of Table 7. Each line crosses zero at its DT–specific turning point. As DT increases, the zero crossing moves to the right and the marginal effect at the upper end of the CR distribution declines. Because the vertical axis is a marginal effect rather than the level of PHD, negative values indicate a negative conditional slope and do not imply that PHD itself is negative.

**Figure 5:**
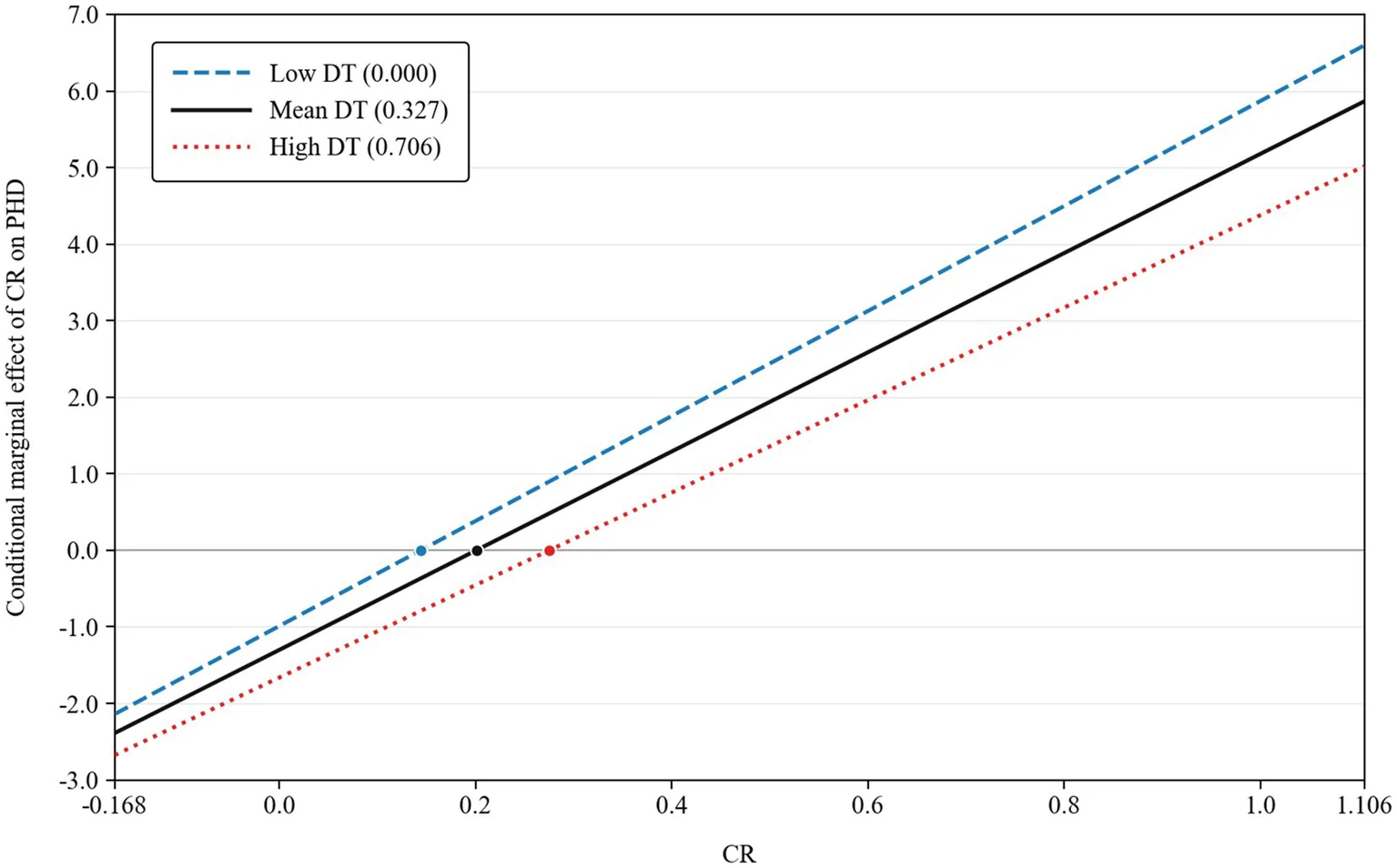
Conditional marginal effect of CR on PHD at DT-specific levels.

### 5.4 Heterogeneity effects

Table 9 reports the panel quantile model estimates. The CR coefficient becomes more negative from the 10th to the 90th quantile, while the CR^2^ coefficient becomes progressively more positive. Because differences in statistical significance do not establish coefficient differences, we supplement these estimates with clustered–bootstrap cross–quantile Wald tests reported in Table 10.

**Table 9:** Panel quantile regression results.

	(1)	(2)	(3)	(4)	(5)
	10th	25th	50th	75th	90th
CR	−0.028^***^ (0.008)	−0.051^***^ (0.006)	−0.088^***^ (0.005)	−0.142^***^ (0.009)	−0.210^***^ (0.016)
CR^2^	0.009 (0.012)	0.037^***^ (0.009)	0.081^***^ (0.008)	0.146^***^ (0.014)	0.227^***^ (0.024)
Control variables	Yes	Yes	Yes	Yes	Yes
$\mu_{i}$	Yes	Yes	Yes	Yes	Yes
$\mu_{t}$	Yes	Yes	Yes	Yes	Yes
*N*	27,151	27,151	27,151	27,151	27,151

Standard errors are reported in parentheses. ^*^ *p* < 0.1, ^**^ *p* < 0.05, ^***^ *p* < 0.01.

**Table 10:** Formal cross-quantile coefficient-equality tests.

Null hypothesis	Wald chi-square	df	*p*-value
CR coefficients are equal across all quantiles	118.64	4	<0.001
CR^2^ coefficients are equal across all quantiles	74.28	4	<0.001
CR and CR^2^ are jointly equal across all quantiles	156.37	8	<0.001
Joint equality: 10^th^ = 90^th^	92.41	2	<0.001
Joint equality: 25^th^ = 75^th^	58.76	2	<0.001
Joint equality: 10^th^ = 25^th^	7.96	2	0.019
Joint equality: 75^th^ = 90^th^	16.83	2	<0.001

Tests use 1,000 firm-clustered bootstrap replications.

The omnibus tests of Table 10 reject equality of the CR coefficients across all quantiles (Wald chi-square = 118.64, *p* < 0.001), equality of the CR^2^ coefficients (Wald chi-square = 74.28, *p* < 0.001), and their joint equality (Wald chi-square = 156.37, *p* < 0.001). Pairwise joint tests also reject equality between the 10^th^ and 90^th^ quantiles and between the 25th and 75th quantiles. The increasing magnitude of both nonlinear terms is therefore supported by formal cross–quantile comparisons rather than by differences in significance alone.

Figure 6 provides a graphical summary of the quantile–specific coefficients and their confidence intervals. Combined with the formal tests, the results indicate that the CR–PHD profile changes across the conditional PHD distribution. The nonlinear response is comparatively weak at the lower tail and becomes more pronounced toward the middle and upper quantiles, supporting *Hypothesis 3*. This interpretation concerns differences in disclosure responses across the conditional distribution of PHD and does not imply differences in objective population health.

**Figure 6:**
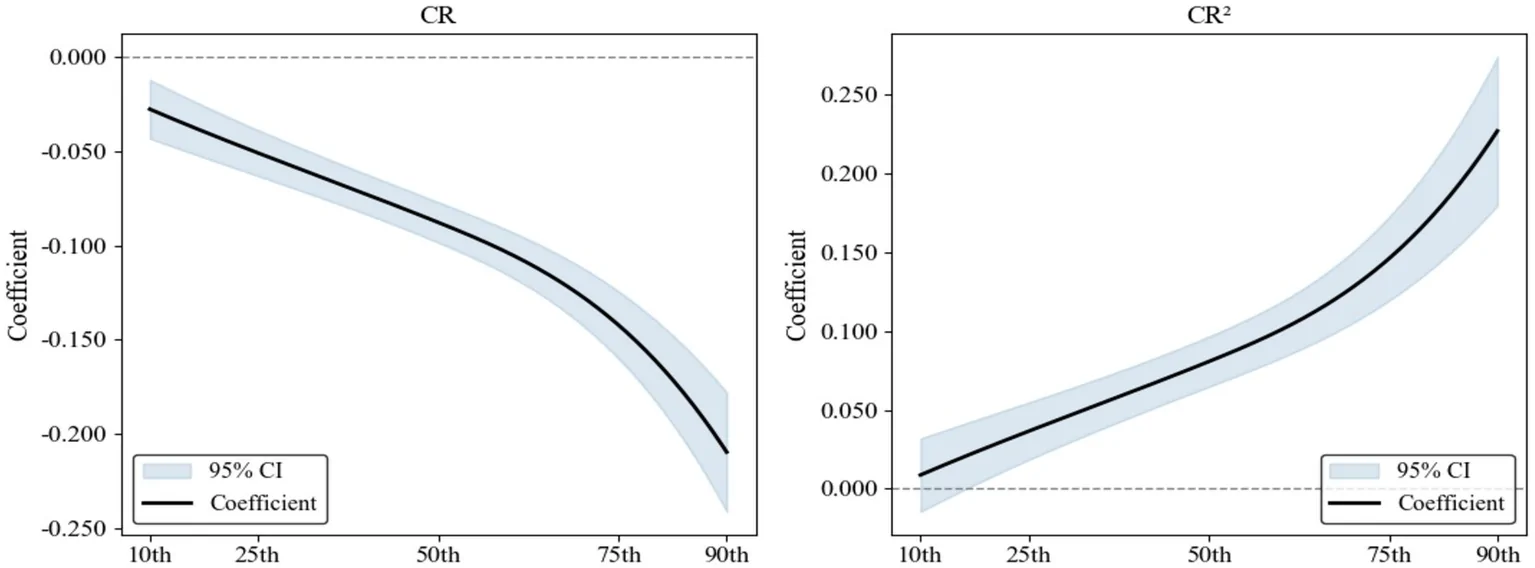
Panel quantile regression coefficient chart.

Table 11 reports subgroup estimates by marketization, marginal profit, and return on assets. To test whether the subgroup coefficients differ statistically, we additionally estimate pooled models containing interactions between the group indicators, CR, and CR^2^. Table 12 reports Wald tests of the joint equality of the CR and CR^2^ coefficients across and between groups.

**Table 11:** Heterogeneity test.

Variable	Marketization level			Marginal profit margin			Return on assets			Sub-dictionaries	
	Low	Medium	High	Low	Medium	High	Low	Medium	High	PHAD	AHRD
CR	−0.018^***^ (0.006)	−0.150^***^ (0.017)	−0.107^***^ (0.025)	−0.086^***^ (0.016)	−0.106^***^ (0.025)	−0.043^***^ (0.015)	−0.069^***^ (0.016)	−0.125^***^ (0.016)	−0.092^***^ (0.016)	−0.055^***^ (0.009)	−0.030 (0.116)
CR^2^	−0.009 (0.020)	0.041^***^ (0.008)	0.076^***^ (0.012)	0.074 (0.117)	0.035^**^ (0.017)	0.088^***^ (0.012)	0.016 (0.021)	0.059^***^ (0.020)	0.072^***^ (0.013)	0.049^***^ (0.010)	0.001 (0.024)
Control variables	Yes	Yes	Yes	Yes	Yes	Yes	Yes	Yes	Yes	Yes	Yes
$\mu_{i}$	Yes	Yes	Yes	Yes	Yes	Yes	Yes	Yes	Yes	Yes	Yes
$\mu_{t}$	Yes	Yes	Yes	Yes	Yes	Yes	Yes	Yes	Yes	Yes	Yes
*Adj R^2^*	0.338	0.320	0.387	0.507	0.349	0.550	0.337	0.472	0.441	0.518	0.430
*N*	8,948	8,872	9,058	8,976	9,026	9,023	8,973	9,170	9,008	27,006	26,842

Standard errors are reported in parentheses. ^*^ *p* < 0.1, ^**^ *p* < 0.05, ^***^ *p* < 0.01.

**Table 12:** Formal cross-group coefficient-equality tests.

Grouping variable/hypothesis	Wald chi-square	df	*p*-value
Marketization: joint equality across three groups	67.42	4	<0.001
Marketization: Low = Medium	52.18	2	<0.001
Marketization: Low = High	15.72	2	<0.001
Marketization: Medium = High	6.54	2	0.038
Marginal profit: joint equality across three groups	5.93	4	0.204
Marginal profit: Low = Medium	1.28	2	0.527
Marginal profit: Low = High	4.12	2	0.128
Marginal profit: Medium = High	5.24	2	0.073
ROA: joint equality across three groups	11.38	4	0.023
ROA: Low = Medium	7.02	2	0.030
ROA: Low = High	8.14	2	0.017
ROA: Medium = High	2.08	2	0.354

Tests are based on pooled interaction models and firm-clustered standard errors.

For the marketization groups, the omnibus test rejects joint coefficient equality (Wald chi-square = 67.42, *p* < 0.001). The pairwise tests also identify differences between the low– and medium–marketization groups, the low– and high–marketization groups, and the medium– and high–marketization groups. These formal comparisons support *Hypothesis 4* and indicate that the nonlinear CR–PHD relationship varies with the regional market environment.

For marginal profit, the subgroup estimates display different signs and significance levels, but the omnibus equality test is not significant (Wald chi-square = 5.93, *p* = 0.204). None of the pairwise joint tests is significant at the 5% level. The subgroup patterns are therefore treated as descriptive, and *Hypothesis 5* is not supported by the formal coefficient difference tests.

For return on assets, the omnibus test rejects equality of the nonlinear coefficients across the three groups (Wald chi-square = 11.38, *p* = 0.023). The low–ROA group differs from both the medium– and high–ROA groups, whereas the difference between the medium– and high–ROA groups is not statistically significant. The evidence therefore supports heterogeneity between financially weaker and stronger firms, while providing no basis for ranking the medium– and high–ROA coefficients. This result provides qualified support for *Hypothesis 6*.

The formal tests identify robust cross–quantile variation and statistically significant subgroup differences for marketization and ROA. By contrast, the apparent variation across marginal profit groups is not confirmed statistically. The revised interpretation therefore distinguishes descriptive coefficient patterns from formally supported heterogeneity.

To distinguish disclosures with different substantive meanings, we divide the original health related dictionary into two directional sub–dictionaries. The first captures adverse health–risk disclosure (AHRD), referring to firms’ disclosure of health hazards, injuries, diseases, and safety incidents. Unambiguously adverse expressions include occupational disease and work–related injury. The second captures protective health action disclosure (PHAD), referring to preventive, protective, and socially beneficial health related activities. Unambiguously action–oriented expressions include epidemic prevention and medical donations.

Several general expressions, including occupational health, employee health, workplace safety, product safety, community health, and environmental health, do not have an inherent direction. We therefore classify these expressions according to their sentence level context. An occurrence is assigned to AHRD when the same sentence contains adverse contextual cues, such as disease, injury, accident, poisoning, hazard, violation, complaint, recall, compensation, fatality, or harmful exposure. It is assigned to PHAD when the sentence contains action–oriented cues, such as prevention, protection, training, monitoring, medical examination, certification, investment, emergency response, improvement, donation, or assistance. Occurrences without sufficient contextual information are excluded from the directional sub–indices to avoid imposing an arbitrary interpretation. For firm i in year t, the two sub–indices are calculated as follows: AHRD is measured as the number of risk related occurrences per 10,000 words in each annual report, while PHAD is measured as the number of action related occurrences per 10,000 words. The aggregate CPHD measure is calculated as the sum of AHRD and PHAD. A higher CPHD value indicates that health related issues occupy a greater share of the firm’s annual report disclosure, but does not by itself indicate better or worse public health outcomes. A higher AHRD value indicates greater disclosure of adverse health risks or incidents, whereas a higher PHAD value indicates greater disclosure of preventive, protective, or contributory health actions.

To address the possibility that the aggregate CPHD index combines disclosures with different substantive meanings, we re–estimate the benchmark model using AHRD and PHAD as separate dependent variables. The results are reported in the last two columns of Table 11.

When PHAD is used as the dependent variable, the coefficient of CR is negative and statistically significant (−0.055, *p* < 0.001), whereas the coefficient of CR squared is positive and statistically significant (0.049, *p* < 0.001). This further confirms the presence of a U–shaped relationship. Thus, the nonlinear relationship remains after adverse health event expressions are removed from the dependent variable. This finding indicates that the benchmark result is not mechanically driven by combining adverse events with protective health activities. Instead, firms initially reduce or postpone health related protective actions under moderate climate risk pressure, but increase preventive investment, employee protection, emergency response, and community health engagement once climate risk becomes sufficiently salient.

For AHRD, the coefficient of CR is insignificant, while the quadratic term is insignificant. This result should be interpreted as a change in the disclosure salience of health risks and incidents, rather than as direct evidence of deterioration in population health. The different patterns obtained for AHRD and PHAD demonstrate that the two components capture conceptually distinct dimensions of corporate health-related disclosure and support the construct validity of the revised measure.

### 5.5 Construct validity and external validation

Because CR, PHD, and DT are all constructed from corporate annual reports, their observed relationships may partly reflect common variation in disclosure intensity, reporting style, ESG orientation, or managerial attention. We therefore conduct a series of content-validity and external validation tests. These tests assess whether the textual measures capture their proposed constructs rather than firms’ general propensity to provide extensive annual report disclosure.

First, we assess the content validity of PHD and its two components. A random sample of 600 sentences containing health related expressions is independently coded by two researchers. Each sentence is classified as adverse health-risk disclosure (AHRD), protective health-action disclosure (PHAD), or directionally ambiguous disclosure. The two coders reach an agreement rate of 90.3%, with a Cohen’s kappa coefficient of 0.840, indicating a high level of inter–coder reliability. Disagreements are resolved through discussion, and expressions lacking sufficient contextual information are excluded from the directional sub–indices.

Second, we validate AHRD and PHAD using information obtained independently of the annual report texts. PHAD is matched with external records of occupational health and safety certification, medical and charitable donations, employee health programs, and other verifiable health protection activities. AHRD is matched with externally recorded work safety accidents, occupational health violations, administrative penalties, and product safety incidents. All textual and external indicators are standardized before estimation. The validation models include firm and year fixed effects, the baseline control variables, and standard errors clustered at the firm level.

The results reported in Table 13 support the expected directional relationships. PHAD is positively associated with the external health action indicator, with a standardized coefficient of 0.126 and a standard error of 0.028 (*p* < 0.01). AHRD is positively associated with the external health risk and incident indicator, with a coefficient of 0.104 and a standard error of 0.031 (*p* < 0.01). In contrast, the cross–construct associations are small and statistically insignificant. These results provide evidence of both convergent and discriminant validity: PHAD captures firms’ disclosed health protection activities, whereas AHRD captures the disclosed salience of adverse health risks and incidents. The aggregate PHD measure is therefore interpreted only as the overall salience of health–related issues in corporate disclosure, rather than as an indicator of better or worse population health.

**Table 13:** Construct validity and external validation of the text-based measures.

Panel A. Manual validation of the PHD dictionary				
Classification	Number of sentences	Precision	Recall	F1 score
AHRD	210	0.881	0.857	0.869
PHAD	248	0.914	0.891	0.902
Directionally ambiguous disclosure	142	0.838	0.817	0.827
Total	600			
Overall agreement rate	90.3%			
Cohen’s kappa	0.840			

Panel B. External validation of AHRD and PHAD				
Variables	External health-action index	Medical and charitable donation intensity	External health-risk incident index	Safety penalty intensity
PHAD	0.126^***^ (0.028)	0.083^***^ (0.021)	0.014 (0.018)	0.006 (0.017)
AHRD	0.011 (0.016)	0.008 (0.015)	0.104^***^ (0.031)	0.077^**^ (0.034)
Control variables	Yes	Yes	Yes	Yes
$\mu_{i}$	Yes	Yes	Yes	Yes
$\mu_{t}$	Yes	Yes	Yes	Yes
*Adj R^2^*	0.436	0.401	0.372	0.358
*N*	18,642	16,908	20,315	19,884

Panel C. External validation of CR and DT				
Variables	Physical climate-risk index	Extreme-weather exposure	Digital patent intensity	Software copyright intensity
Text-based CR	0.286^***^ (0.024)	0.219^***^ (0.027)		
Text-based DT			0.337^***^ (0.030)	0.241^***^ (0.034)
Control variables	Yes	Yes	Yes	Yes
$\mu_{i}$	Yes	Yes	Yes	Yes
$\mu_{t}$	Yes	Yes	Yes	Yes
*Adj R^2^*	0.512	0.487	0.544	0.518
*N*	27,418	27,418	24,736	23,984

Standard errors are reported in parentheses. ^*^p < 0.1, ^**^p < 0.05, ^***^p < 0.01.

Third, we externally validate the textual measures of CR and DT. Text–based CR is compared with a physical climate risk indicator constructed from independently recorded extreme temperatures, floods, droughts, typhoons, and other weather-related events in the cities where firms are registered or operate. Text–based DT is compared with external indicators based on digital invention patents, software copyrights, and verifiable information technology investment. As reported in Panel C of Table 13, text–based CR is positively associated with the physical climate risk index and extreme weather exposure, while text–based DT is positively associated with digital patent and software copyright intensity. The magnitudes are moderate rather than excessively high, suggesting that the textual measures capture related constructs without being mechanical replicas of the external indicators. Taken together, the manual coding and external validation results support the construct validity of AHRD, PHAD, CR, and DT.

## 6 Conclusion and suggestion

### 6.1 Conclusion

Using panel data of Chinese listed firms from 2000 to 2024, this study examines how CR affects PHD and whether this relationship is conditioned by DT, PHD distribution, marketization level, and firm financial capacity. The empirical evidence yields several findings.

First, CR has a significant nonlinear association with PHD. The baseline estimates and the U–test indicate a negative marginal slope at the lower bound and a positive marginal slope at the upper bound of the observed CR distribution. The implied turning point is 0.837 on the CR scale and lies within the sample range. Because PHD measures the salience of public health–related disclosure in corporate annual reports, this result indicates that such disclosure initially decreases and subsequently increases as corporate climate risk salience rises; it should not be interpreted as a corresponding change in objective population health outcomes.

Second, DT has a conditional moderating influence on the CR–PHD relationship. Its association with PHD is positive at relatively low CR levels but diminishes as CR increases. Higher DT also shifts the turning point to the right and reduces the positive marginal effect of CR on the upper branch of the U–shaped relationship. These results suggest that DT strengthens firms’ routine information and disclosure capacity while reducing their reliance on sharp, climate risk induced adjustments in PHD. This interpretation concerns corporate disclosure behavior and does not imply that DT weakens actual adaptation investment or environmental governance.

Third, clustered–bootstrap Wald tests confirm that the CR and CR^2^ coefficients differ jointly across the conditional PHD distribution. The nonlinear relationship is relatively weak at the lower tail and becomes more pronounced at the middle and upper quantiles. This conclusion is based on formal cross–quantile tests rather than comparisons of significance levels.

Fourth, pooled interaction models and cross–group Wald tests identify significant differences across marketization and ROA groups. The corresponding tests do not establish statistically significant differences across marginal profit groups, so those subgroup patterns are interpreted as descriptive rather than confirmatory evidence of heterogeneity.

It should be pointed out that the results identify a U–shaped association between CR and corporate PHD After the turning point, greater CR is associated with more intensive disclosure of health related risks, responsibilities, and protective actions. This finding is consistent with the possibility that highly salient climate risk increases stakeholder scrutiny and firms’ incentives to communicate their responses. It should not, however, be interpreted as evidence that high climate risk automatically improves corporate adaptation, environmental quality, or objective public-health outcomes. It needs to be acknowledged that a limitation of this study is that it does not directly observe firms’ climate-adaptation expenditure, environmental improvements, or substantive governance responses. Accordingly, the mechanisms proposed for the upward portion of the U–shaped relationship remain theoretically motivated interpretations rather than directly identified causal channels. Future research could link PHD with independently observed adaptation investment, environmental certification, green technological innovation, pollution reduction, or formal environmental governance arrangements to test these mechanisms more directly.

### 6.2 Suggestion

These findings provide several policy implications. First, CR governance should be threshold-oriented rather than uniformly designed. For firms below the turning point, policy should focus on reducing climate–risk exposure, strengthening emissions control, and preventing climate pressure from crowding out health–related disclosure and action of PHD. For firms above the turning point, regulation should further guide adaptive investment, green innovation, and climate–risk disclosure so that the emerging disclosure response can be consolidated.

Second, DT should be integrated into substantive governance processes rather than treated as a standalone technological upgrade. Digital platforms should be used to improve climate–risk monitoring, environmental information disclosure, occupational health protection, and early–warning systems for public health risks. The results do not imply that digitalization uniformly increases PHD or weakens substantive climate adaptation. Firms should use digital systems to institutionalize continuous climate- and health-related monitoring and disclosure, while regulators should evaluate the substantive actions underlying such disclosures rather than relying solely on changes in disclosure frequency.

Third, greater policy support should be directed toward firms with relatively low PHD intensity, low–marketization regions, and financially constrained firms, since these groups are less able to transform CR pressure into adaptive responses. Targeted fiscal support, green finance access, technical assistance, and disclosure standards are therefore necessary. Finally, regulators should encourage firms with stronger profitability and higher operational efficiency to take the lead in climate adaptation and health–oriented environmental governance, while preventing financially weaker firms from falling into a cycle of climate exposure, environmental damage, and declining PHD.

## Data Availability

The original contributions presented in the study are included in the article/Supplementary material, further inquiries can be directed to the corresponding author.
