# Supplementary material for: Corporate climate risk and public health disclosure: does digital transformation matter?
Source: Front Public Health. 2026 Sep 23;14:1954722. doi: 10.3389/fpubh.2026.1954722 (PMC13642152; doi:10.3389/fpubh.2026.1954722)
Supplement: Supplementary file 1 [file Supplementary_file_1.docx]

**Appendix: Keyword dictionaries**

All annual reports are converted into machine-readable text and standardized before dictionary matching. Traditional Chinese characters are converted into simplified Chinese, English expressions are converted to a consistent case, and punctuation, page numbers, tables of contents, headers, footers, and non-textual symbols are removed. Chinese word segmentation is conducted using a customized dictionary containing all multi-character expressions listed above. Longer expressions are matched before shorter terms, and each textual span is counted only once within the same dictionary dimension. Dictionary frequencies are normalized by the valid total word count of each annual report.

| **Table S1.** Public health disclosure dictionary | | | | |  |
| --- | --- | --- | --- | --- | --- |
| Dimension or classification | | No. | | Keyword or phrase |  |
| Adverse health-risk disclosure (AHRD) | | 1 | | occupational disease |  |
|  |  | 2 | | occupational injury |  |
|  |  | 3 | | work-related injury |  |
|  |  | 4 | | work-related accident |  |
|  |  | 5 | | production safety accident |  |
|  |  | 6 | | safety accident |  |
|  |  | 7 | | personal injury or death |  |
|  |  | 8 | | employee casualty |  |
|  |  | 9 | | work-related fatality |  |
|  |  | 10 | | poisoning accident |  |
|  |  | 11 | | occupational poisoning |  |
|  |  | 12 | | health hazard |  |
|  |  | 13 | | harmful exposure |  |
|  |  | 14 | | dust hazard |  |
|  |  | 15 | | noise hazard |  |
|  |  | 16 | | radiation hazard |  |
|  |  | 17 | | infectious-disease outbreak |  |
|  |  | 18 | | epidemic infection |  |
|  |  | 19 | | employee infection |  |
|  |  | 20 | | food-safety incident |  |
|  |  | 21 | | product-safety incident |  |
|  |  | 22 | | product recall |  |
|  |  | 23 | | health-related complaint |  |
|  |  | 24 | | health impairment |  |
|  |  | 25 | | medical compensation |  |
|  |  | 26 | | safety-related penalty |  |
|  |  | 27 | | occupational-health violation |  |
|  |  | 28 | | public-health emergency |  |
|  |  | 29 | | epidemic outbreak |  |
|  |  | 30 | | hazardous operation |  |
| Protective health-action disclosure (PHAD) | | 31 | | occupational-health management |  |
|  |  | 32 | | occupational-hygiene management |  |
|  |  | 33 | | occupational-health protection |  |
|  |  | 34 | | employee-health management |  |
|  |  | 35 | | employee-health protection |  |
|  |  | 36 | | health examination |  |
|  |  | 37 | | occupational-health examination |  |
|  |  | 38 | | mental-health service |  |
|  |  | 39 | | health consultation |  |
|  |  | 40 | | health training |  |
|  |  | 41 | | production-safety training |  |
|  |  | 42 | | occupational-hygiene training |  |
|  |  | 43 | | labor protection |  |
|  |  | 44 | | personal protection |  |
|  |  | 45 | | personal protective equipment |  |
|  |  | 46 | | workplace monitoring |  |
|  |  | 47 | | occupational-hazard monitoring |  |
|  |  | 48 | | epidemic prevention and control |  |
|  |  | 49 | | disease prevention |  |
|  |  | 50 | | emergency epidemic prevention |  |
|  |  | 51 | | vaccination |  |
|  |  | 52 | | medical relief |  |
|  |  | 53 | | medical assistance |  |
|  |  | 54 | | medical donation |  |
|  |  | 55 | | epidemic-relief donation |  |
|  |  | 56 | | health-related philanthropy |  |
|  |  | 57 | | community health service |  |
|  |  | 58 | | public-health assistance |  |
|  |  | 59 | | emergency rescue |  |
|  |  | 60 | | health promotion |  |
|  |  | 61 | | product-safety management |  |
|  |  | 62 | | food-safety management |  |
|  |  | 63 | | employee medical insurance |  |
|  |  | 64 | | supplementary medical insurance |  |
|  |  | 65 | | health and safety certification |  |
| Directionally neutral health expression | | 66 | | occupational health |  |
|  |  | 67 | | occupational hygiene |  |
|  |  | 68 | | employee health |  |
|  |  | 69 | | workplace safety |  |
|  |  | 70 | | production safety |  |
|  |  | 71 | | product safety |  |
|  |  | 72 | | food safety |  |
|  |  | 73 | | community health |  |
|  |  | 74 | | public health |  |
|  |  | 75 | | environmental health |  |
|  |  | 76 | | health and safety |  |
| Note: Direct AHRD and PHAD expressions are classified according to their inherent meaning. Directionally neutral expressions are classified using cues in the same sentence. Adverse cues are: disease; injury; accident; poisoning; hazard; violation; complaint; recall; compensation; death; infection; outbreak; harmful exposure; exposure; penalty; damage. Protective-action cues are: prevention; protection; training; monitoring; medical examination; certification; investment; governance; control; relief; donation; assistance; improvement; emergency response; promotion; safeguard. If both sets of cues occur and direction cannot be determined reliably, the occurrence is retained in aggregate PHD but excluded from the directional sub-indices. | | | | |  |
| **Table S2.** Climate risk dictionary | | | | | |
| Dimension or classification | No. | | Keyword or phrase | | |
| Acute physical risk | 1 | | extreme weather | | |
|  | 2 | | extreme climate event | | |
|  | 3 | | heavy rainfall | | |
|  | 4 | | extreme rainfall | | |
|  | 5 | | flood | | |
|  | 6 | | flooding | | |
|  | 7 | | urban waterlogging | | |
|  | 8 | | typhoon | | |
|  | 9 | | tropical cyclone | | |
|  | 10 | | storm surge | | |
|  | 11 | | high temperature | | |
|  | 12 | | extreme heat | | |
|  | 13 | | heatwave | | |
|  | 14 | | drought | | |
|  | 15 | | cold wave | | |
|  | 16 | | freezing event | | |
|  | 17 | | blizzard | | |
|  | 18 | | wildfire | | |
|  | 19 | | forest fire | | |
|  | 20 | | sandstorm | | |
| Chronic physical risk | 21 | | climate change | | |
|  | 22 | | global warming | | |
|  | 23 | | climate warming | | |
|  | 24 | | greenhouse effect | | |
|  | 25 | | temperature rise | | |
|  | 26 | | change in precipitation | | |
|  | 27 | | abnormal precipitation | | |
|  | 28 | | sea-level rise | | |
|  | 29 | | water scarcity | | |
|  | 30 | | desertification | | |
|  | 31 | | ecological degradation | | |
|  | 32 | | climate anomaly | | |
| Policy and legal transition risk | 33 | | carbon peaking | | |
|  | 34 | | carbon neutrality | | |
|  | 35 | | dual-carbon targets | | |
|  | 36 | | carbon emission allowance | | |
|  | 37 | | emissions trading scheme | | |
|  | 38 | | carbon trading | | |
|  | 39 | | carbon market | | |
|  | 40 | | carbon quota | | |
|  | 41 | | carbon tax | | |
|  | 42 | | carbon pricing | | |
|  | 43 | | carbon border adjustment mechanism | | |
|  | 44 | | greenhouse-gas emissions standard | | |
|  | 45 | | carbon emissions regulation | | |
|  | 46 | | climate policy | | |
|  | 47 | | low-carbon policy | | |
|  | 48 | | emissions-reduction policy | | |
|  | 49 | | mandatory emissions reduction | | |
|  | 50 | | emissions constraint | | |
|  | 51 | | climate litigation | | |
|  | 52 | | climate compliance | | |
| Market, technology, and reputational transition risk | 53 | | low-carbon transition | | |
|  | 54 | | energy transition | | |
|  | 55 | | transition risk | | |
|  | 56 | | carbon-abatement cost | | |
|  | 57 | | emissions-reduction cost | | |
|  | 58 | | carbon-asset risk | | |
|  | 59 | | carbon-price volatility | | |
|  | 60 | | fossil-fuel phase-out | | |
|  | 61 | | clean-energy substitution | | |
|  | 62 | | low-carbon technology substitution | | |
|  | 63 | | green-technology substitution | | |
|  | 64 | | stranded asset | | |
|  | 65 | | high-carbon asset | | |
|  | 66 | | supply-chain decarbonization | | |
|  | 67 | | supply-chain carbon risk | | |
|  | 68 | | climate-related disclosure | | |
|  | 69 | | carbon disclosure | | |
|  | 70 | | climate-related reputational risk | | |
|  | 71 | | customer preference for low-carbon products | | |
|  | 72 | | investor attention to climate risk | | |
| Note: CR dictionary contains 72 core phrases. General transition-risk expressions are retained only when at least one risk-context cue appears in the same sentence. The context cues are: risk; shock; pressure; uncertainty; exposure; loss; cost; constraint; compliance; penalty; impact; threat; vulnerability. Longer phrases are matched before shorter nested phrases. | | | | | |

| **Table S3.** Digital transformation dictionary | | |
| --- | --- | --- |
| Dimension or classification | No. | Keyword or phrase |
| Digital technologies | 1 | digitalization |
|  | 2 | digital technology |
|  | 3 | digital economy |
|  | 4 | artificial intelligence |
|  | 5 | AI |
|  | 6 | machine learning |
|  | 7 | deep learning |
|  | 8 | neural network |
|  | 9 | natural language processing |
|  | 10 | computer vision |
|  | 11 | knowledge graph |
|  | 12 | big data |
|  | 13 | data mining |
|  | 14 | data analytics |
|  | 15 | business intelligence |
|  | 16 | cloud computing |
|  | 17 | cloud platform |
|  | 18 | cloud service |
|  | 19 | edge computing |
|  | 20 | blockchain |
|  | 21 | distributed ledger |
|  | 22 | Internet of Things |
|  | 23 | industrial Internet |
|  | 24 | mobile Internet |
|  | 25 | 5G |
|  | 26 | fifth-generation mobile communications |
|  | 27 | digital twin |
|  | 28 | virtual reality |
|  | 29 | augmented reality |
|  | 30 | metaverse |
|  | 31 | robotics |
|  | 32 | intelligent robot |
|  | 33 | robotic process automation |
|  | 34 | automated algorithm |
|  | 35 | intelligent algorithm |
|  | 36 | enterprise data platform |
|  | 37 | enterprise technology platform |
|  | 38 | computing power |
|  | 39 | data factor |
|  | 40 | data asset |
|  | 41 | privacy-preserving computing |
|  | 42 | cybersecurity |
|  | 43 | information security |
|  | 44 | data security |
| Digital organizational empowerment | 45 | digital transformation |
|  | 46 | digital strategy |
|  | 47 | digital management |
|  | 48 | digital governance |
|  | 49 | digital organization |
|  | 50 | digital operations |
|  | 51 | digital platform |
|  | 52 | digital system |
|  | 53 | digital infrastructure |
|  | 54 | informatization |
|  | 55 | information system |
|  | 56 | information management system |
|  | 57 | management information system |
|  | 58 | enterprise resource planning |
|  | 59 | ERP |
|  | 60 | manufacturing execution system |
|  | 61 | MES |
|  | 62 | customer relationship management |
|  | 63 | CRM |
|  | 64 | supply-chain management system |
|  | 65 | SCM |
|  | 66 | office automation |
|  | 67 | OA |
|  | 68 | decision-support system |
|  | 69 | intelligent decision-making |
|  | 70 | data-driven management |
|  | 71 | data governance |
|  | 72 | master-data management |
|  | 73 | data warehouse |
|  | 74 | data center |
|  | 75 | shared-service center |
|  | 76 | financial shared service |
|  | 77 | business-finance integration |
|  | 78 | process digitalization |
|  | 79 | organizational digitalization |
|  | 80 | digital talent |
|  | 81 | chief data officer |
|  | 82 | information-technology investment |
|  | 83 | software investment |
|  | 84 | digital investment |
| Digital business applications | 85 | intelligent manufacturing |
|  | 86 | digital manufacturing |
|  | 87 | smart factory |
|  | 88 | digital factory |
|  | 89 | unmanned factory |
|  | 90 | intelligent production |
|  | 91 | flexible manufacturing |
|  | 92 | automated production |
|  | 93 | intelligent equipment |
|  | 94 | smart warehousing |
|  | 95 | smart logistics |
|  | 96 | digital supply chain |
|  | 97 | smart supply chain |
|  | 98 | supply-chain collaboration |
|  | 99 | electronic commerce |
|  | 100 | e-commerce platform |
|  | 101 | online platform |
|  | 102 | Internet platform |
|  | 103 | mobile application |
|  | 104 | APP |
|  | 105 | mini program |
|  | 106 | online service |
|  | 107 | digital service |
|  | 108 | digital marketing |
|  | 109 | precision marketing |
|  | 110 | intelligent marketing |
|  | 111 | user profiling |
|  | 112 | intelligent customer service |
|  | 113 | online customer service |
|  | 114 | mobile payment |
|  | 115 | digital payment |
|  | 116 | smart retail |
|  | 117 | new retail |
|  | 118 | financial technology |
|  | 119 | smart finance |
|  | 120 | remote working |
|  | 121 | video conferencing |
|  | 122 | paperless office |
|  | 123 | smart energy |
|  | 124 | smart mine |
|  | 125 | intelligent transportation |
|  | 126 | smart healthcare |
|  | 127 | telemedicine |
|  | 128 | intelligent operations and maintenance |
|  | 129 | predictive maintenance |
|  | 130 | intelligent quality inspection |
|  | 131 | machine-vision inspection |
|  | 132 | intelligent risk control |
|  | 133 | digital risk control |
|  | 134 | digital procurement |
|  | 135 | electronic procurement |
|  | 136 | platform-based operations |
|  | 137 | industrial Internet platform |
|  | 138 | Internet of Vehicles |
|  | 139 | smart industrial park |
| Note: DT dictionary contains 139 terms: 44 digital-technology terms, 40 digital organizational empowerment terms, and 55 digital business application terms. English abbreviations are matched without case sensitivity. A longest-match rule prevents duplicate counting of nested expressions. | | |
